# Knowledge, attitudes and practices of malaria in Colombia

**DOI:** 10.1186/1475-2875-13-165

**Published:** 2014-05-01

**Authors:** David A Forero, Pablo E Chaparro, Andres F Vallejo, Yoldy Benavides, Juan B Gutiérrez, Myriam Arévalo-Herrera, Sócrates Herrera

**Affiliations:** 1 Malaria Vaccine and Drug Development Center, Cali, Colombia; 2 National Institute of Health of Colombia, Bogotá, Colombia; 3 Institute of Bioinformatics & Department of Mathematics, University of Georgia, Athens, GA, USA; 4 Caucaseco Scientific Research Center, Cali, Colombia; 5 School of Health, Universidad del Valle, Cali, Colombia; 6 Latin American Center for Malaria Research, Cali, Colombia

## Abstract

**Background:**

Although Colombia has witnessed an important decrease in malaria transmission, the disease remains a public health problem with an estimated ~10 million people currently living in areas with malaria risk and ~61,000 cases reported in 2012. This study aimed to determine and compare the level of knowledge, attitudes and practices (KAP) about malaria in three endemic communities of Colombia to provide the knowledge framework for development of new intervention strategies for malaria elimination.

**Methods:**

A cross-sectional KAP survey was conducted in the municipalities of Tierralta, Buenaventura and Tumaco, categorized according to high risk (HR) and moderate risk (MR) based on the annual parasite index (API). Surveys were managed using REDCap and analysed using MATLAB and GraphPad Prism.

**Results:**

A total of 267 residents, mostly women (74%) were surveyed. Although no differences were observed on the knowledge of classical malaria symptoms between HR and MR regions, significant differences were found in knowledge and attitudes about transmission mechanisms, anti-malarial use and malaria diagnosis. Most responders in both regions (93.5% in MR, and 94.3% in HR areas) indicated use of insecticide-treated nets (ITNs) to protect themselves from malaria, and 75.5% of responders in HR indicated they did nothing to prevent malaria transmission outdoors. Despite a high level of knowledge in the study regions, significant gaps persisted relating to practices. Self-medication and poor adherence to treatment, as well as lack of both indoor and outdoor vector control measures, were significantly associated with higher malaria risk.

**Conclusions:**

Although significant efforts are currently being made by the Ministry of Health to use community education as one of the main components of the control strategy, these generic education programmes may not be applicable to all endemic regions of Colombia given the substantial geographic, ethnic and cultural diversity.

## Background

Malaria causes over 207 million clinical cases and ~627,000 deaths worldwide every year representing an enormous global, social and economic burden [1]. Although, the American continent contributes a minor percentage of these cases, malaria is endemic in 21 countries of the continent with ~469,000 cases reported in 2012, most of them from Brazil (52%) and Colombia (13%) [1]. Several countries of the region, such as Argentina, Belize, Bolivia, Costa Rica, Ecuador, El Salvador, French Guiana, Guatemala, Honduras, Mexico, Nicaragua, Paraguay, and Suriname have experienced a drastic decrease in malaria case incidence (>75%) during the last decade [1]. Even though Colombia has also witnessed an important decrease in transmission, malaria remains a public health problem with an unstable transmission pattern that in 2010 accounted for ~117,000 cases [2] and ~61,000 cases in 2012 [3]. An estimated ~10 million people currently live in areas with variable malaria risk, mainly distributed in the north-western part of the country (departments of Córdoba and Antioquia) and in the western region, along the Pacific coast, comprised of the departments of Chocó, Valle, Cauca and Nariño. In this most endemic area, ~12.5% of the population live in high risk (HR) areas with annual parasite index (API) ≥10, ~20.0% in moderate risk (MR) areas with API values of 1 to 10, and the remaining (67.5%) in low transmission areas (API <1). In addition, there is an extraordinary geographic, ethnic and cultural diversity among these regions [4].

Although previous studies have indicated that malaria risk in both rural and urban endemic areas of Colombia is associated with factors such as age, number of people per household, occupation and level of education [5-8], regional differences in terms of transmission intensities and cultural background have not been determined.

Prevention and control activities developed by the National Malaria Control Programme (NMCP) of Colombia are mainly focused on early diagnosis and prompt treatment; distribution of long-lasting, insecticide-treated nets (LLINs); and education programmes. Despite these activities, the milestones proposed by the Ministry of Health (MoH) for reducing morbidity by 70% and eliminating urban malaria transmission by 2021 [9] have not been reached completely due to factors such as presence of malaria in remote areas with limited access to health and education services, political instability and several other constraints.

Two of the study areas, Tierralta (HR) and Buenaventura (MR), are currently the subject of active control activities developed jointly between the NMCP and a project sponsored by the Global Fund for AIDS, Tuberculosis and Malaria (GFATM) where education is one of the major components. The ongoing education effort consists of distributing booklets, videos, shirts, school bags, and other materials targeting children; and games, murals, and advertisements that target adults.

Because malaria is associated with poverty [10], most endemic countries or regions within a country usually correspond to those communities with the lowest socio-economic status. These are frequently found in regions where malaria control is logistically and economically more challenging due to the limited capacity of local and national governments to invest in health and infrastructure. In these areas, community participation in malaria control and elimination activities is essential to achieve success and sustainability. A community’s commitment to participate in malaria prevention requires a minimal level of education in order to develop an adequate understanding of transmission, and thereby contribute to adapting attitudes towards malaria control/elimination [11-13].

In view of the limited knowledge, attitudes and practices (KAP) studies in Colombia [7,8,14], and the growing national interest in strengthening malaria control activities [9], the purpose of this study was to determine and compare KAP about malaria in the communities of three municipalities of Colombia, and to provide the knowledge framework for development of new intervention strategies for malaria elimination.

## Methods

### Study sites

**Figure 1:**
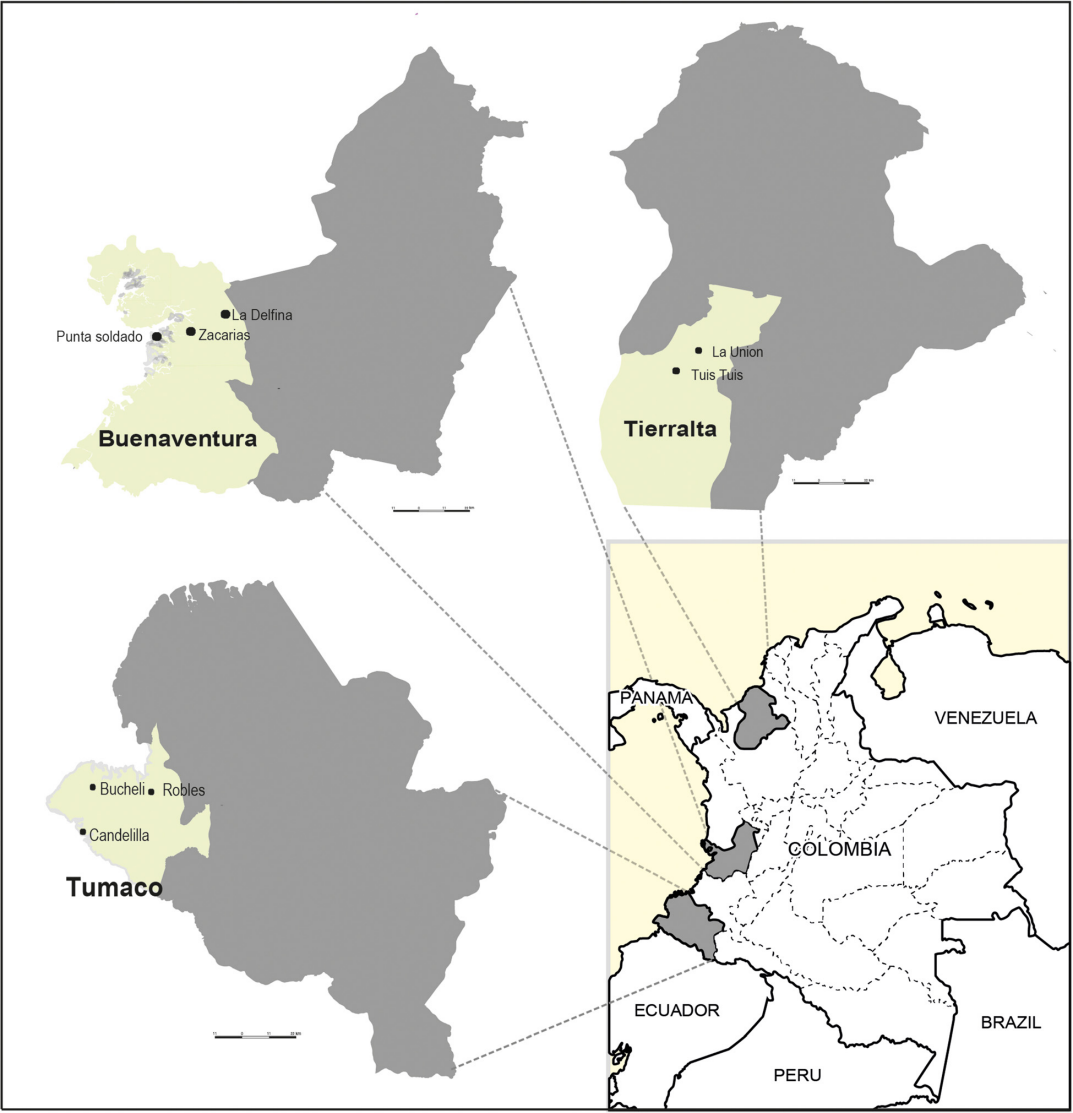
**Study sites.** Map of Colombia showing the location of the sentinel study sites. The insert depicts the location of the study areas within the country.

Sites selected for this survey included Tierralta, Buenaventura and Tumaco (Figure 1). Tierralta is a municipality located in the department of Cordoba in the northwestern part of the country, at 51 m above sea level (masl), 08°10′34″ north latitude and 76°03′46″ west longitude, covering an area of 5,079 sq km, with an average temperature of 27.3°C. Tierralta has a population of ~90,000 inhabitants, 44.4% of which live in rural areas, 55.6% in the urban area, with ~2% indigenous population. The predominant malaria parasite species in the region is *Plasmodium vivax* (82.4%) followed by *Plasmodium falciparum* (17.4%), and 0.2% of mixed malaria infections. The sentinel sites selected in this region were Tuis Tuis and La Union.

Buenaventura is located on the Pacific coast in the department of Valle in the western part of Colombia, at 7 masl, covering an area of 6,078 sq km, with an average temperature of 28°C. The region consists of a tropical rainforest with a relative humidity of 85% and ~8,000 mm of annual rainfall. Its population of ~350,000 inhabitants is predominantly Afro-descendant (72.4%), with 13.4% considered white, 8.5% indigenous, and 5.7% *mestizo*. Malaria occurs throughout the year, with two small seasonal transmission peaks from April to May and September to October [15]. The predominant malaria parasite species in the region is *P. vivax* (85%) followed by *P. falciparum* (15%). The sentinel sites selected in this region were Punta Soldado, Zacarias and La Delfina.

Tumaco is located in the department of Nariño, in the south-western region of the country, near the border with Ecuador, at 2 masl, covering an area of 3,778 sq km, with an average temperature of 28°C. The population of Tumaco, estimated at 187,084 inhabitants, is composed mainly of Afro-descendants (88%). *Plasmodium falciparum* has a prevalence of 79.2% and 20.8% *P. vivax.* The sentinel sites selected in this region were Robles, Candelilla and Bucheli.

The population was divided into two groups according to the API recorded in the last five years (2008–2012), HR areas corresponded to API ≥10, whereas MR were areas with 10 > API > 1. Based on these categories, Tierralta (Córdoba) with API of 44.0 cases/1,000 habitants was considered an HR area, whereas Buenaventura (Valle del Cauca) and Tumaco (Nariño), with API of 6.0 cases/1,000 habitants and 7.7 cases/1,000 habitants, respectively, were considered MR areas.

### Study design and ethical approval

The study corresponded to a cross-sectional survey, conducted between October and December 2011 in eight sentinel sites selected based on API and socio-demographic characteristics. The study was developed in two steps: the first, a door-to-door census of the entire population of each sentinel site; and the second, a survey using a structured questionnaire carried out by trained field workers. The survey was directed to heads of households in a subset of randomly selected houses. The same team of trained physicians accompanied the local teams to all eight sentinel sites. The trial was conducted according to Declaration of Helsinki principles, International Conference on Harmonization, ICH E-6 Guidelines for Good Clinical Practices and all pertinent Colombian regulations. The study protocol was approved by Institutional Review Boards (IRB) of the Malaria Vaccine and Drug Development Center–MVDC (CECIV,Cali) (Number: 009). Written informed consent (IC) was obtained from each volunteer at enrollment.

### KAP evaluation and socio-demographic assessment

The questionnaire had a total of 41 questions divided as follows: 12 questions on knowledge of the malaria vector, symptoms, treatment, diagnosis, and prevention measures; ten questions on attitudes towards transmission, prevention and diagnosis; and, nine questions on practices such as the use of insecticide-treated nets (ITN), and thick blood smear (TBS) diagnosis and treatment. For assessment of socio-demographic characteristics, ten questions on public services, education level, ethnicity, and employment were included.

### Data analysis

Data entry: information was captured in the field on paper-based case report forms (CRF). Data were digitized using REDCap (version 4.1) and imported into MATLAB (version 2011b) for analysis. The number of CRFs per location was: Buenaventura, 105; Tierralta, 53; Tumaco, 109. The MATLAB script used for analysis was provided in Additional file 1.

### Data quality assurance

The data quality assurance (QA) procedure consisted of setting a quality control (QC) sample of size *q* less than the total number *p* of all CRFs for each location. Since during the QC procedure all errors found were corrected, it is possible to calculate the number of iterations of QC needed to bring the error below an acceptable threshold. During the first iteration of QC, $e=\frac{\tilde{q}}{q}$ represents the percentage of error found during a QC procedure, where $\tilde{q}$ is the number of CRFs with at least one error. Assuming a uniform distribution of error, the percentage of total error after finding and correcting errors in the first QC sample of size *q* is $T=\frac{e}{q}\left(p-q\right).$ After *n* iterations of QC, the percentage of total error is $T=\frac{e}{q}\left(p-\mathrm{nq}\right).$ Let *T*_*a*_ represent now the target QA threshold of acceptable error. Then, the number *n* of iterations of QC needed to bring down the error to acceptable levels is $n=\frac{p}{q}\left(1-\frac{T_{a}}{e}\right)$. The maximum permissible error *T*_*a*_ for this study was established at 1%. While this procedure might seem unnecessary for such a small sample, it lays the foundation for more comprehensive studies that will be conducted in the near future as a continuation of the present study.

Using descriptive statistics, the general characteristics of communities, families, and individuals admitted to the study were established. Measures of central tendency and dispersion were calculated for quantitative characteristics, whereas absolute frequencies as well as confidence intervals were used for qualitative characteristics.

For each question, association tests were performed using the Chi-square test with significant differences set at p-value <0.05. Some questions in the CRFs were used to determine the correlation of malaria incidence and KAP. For the purpose of analysis, incidence was estimated taking into account the records of the NMCP, which were based on microscopic analyses of active and passive case detections.

## Results

### Characteristics of respondents and housing

A total of 267 houses inhabited by 1,170 residents were surveyed; 214 houses were located in MR, and 53 in HR. In MR areas (Tumaco and Buenaventura), 13.2% of responders reported at least one malaria episode in the last year in the household, whereas in the HR area (Tierralta) this proportion was found to be 25.2%. This information was used for association analysis.

The surveys were administered to heads of households, who in most cases were women (MR = 75.7%; HR = 67.9%). Most of the participants were housewives (MR = 56.5%; HR = 62.3%); some were students or had other occupations (Table 1). A small proportion of responders (8.9%) in MR had never attended school compared with 17.0% in HR; 47.7% of the participants from MR, and 64.2% from HR had at least a primary education.

**Table 1:** Demographic characteristics of persons in selected households

	**Medium risk**		**High risk**		
	**(n = 214)**		**(n = 53)**		
**Gender**	**n**	**%**	**n**	**%**	**p-value**
**Female**	162	75.7	36	67.9	0.247
**Male**	52	24.3	17	32.1	
**Occupation**					
**Labourer**	10	4.7	14	26.4	0.000
**Farmer**	19	8.9	1	1.9	0.083
**Housewife**	121	56.5	33	62.3	0.450
**Student**	13	6.1	0	0.0	0.065
**House type**					
**Brick**	110	51.4	9	17.0	0.000
**Wooden hut**	75	35.0	1	1.9	0.000
**Shacks**	25	11.7	43	81.1	0.000
**Where is the sanitary service?**					
**Inside the house**	93	43.5	3	5.7	0.000
**Outside the house**	119	55.6	50	94.3	
**Distance source of water**					
**Inside House**	130	60.7	1	1.9	0.000
**Less than 20 m**	26	12.1	15	28.3	0.003
**Between 50 and 100 m**	13	6.1	13	24.5	0.000
**More than 100 m**	5	2.3	23	43.4	0.000
**Source of water**					
**Aqueduct**	133	62.1	1	1.9	0.000
**Well**	23	10.7	19	35.8	0.000
**River**	5	2.3	28	52.8	0.000
**Rainwater**	47	22.0	5	9.4	0.039

**Figure 2:**
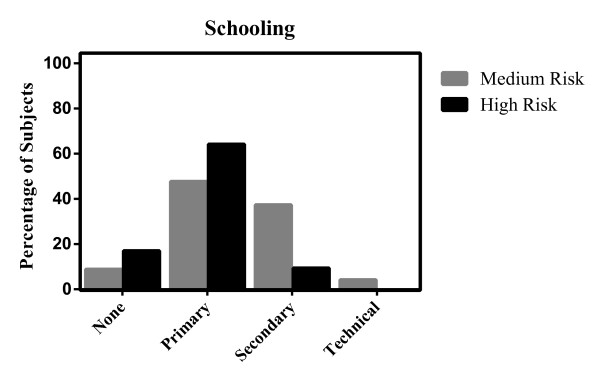
Education.

A higher percentage of the population from MR areas (37.4%) than HR areas (9.4%) had some level of secondary education; 4.2% in MR areas, and 0% in HR areas had technical education (Figure 2). Houses in MR areas were constructed of brick (51.4%) and wood (35.0%); 81.3% had electricity; and 62.1% had a drinking water supply. In contrast, the houses in HR were built of brick (17.0%) and wood (1.9%); 100% had electricity; and only 1.9% of houses had an aqueduct.

### Knowledge

Of the total responders, 86.9% in MR areas and 79.2% in HR areas knew that malaria is acquired by the bite of an infected mosquito and a small proportion (HR = 5.7%) knew that malaria could be transmitted through blood transfusion. In both groups, fever, headache and chills were identified as the first symptoms detected in a person with malaria. Other recognized symptoms were myalgia and arthralgia, weakness and sweating; no significant differences were found between the two groups. Additionally, ~90% in both groups reported to have visited a local clinic with a microscopist or other health officer when they had malaria symptoms (Table 2).

**Table 2:** Malaria knowledge of participants in the selected study households

	**Medium risk**		**High risk**		
	**(n=214)**		**(n = 53)**		
	**n**	**%**	**n**	**%**	**p-value**
**Yes/No questions**					
Does the person know how malaria is transmitted? (Yes)	186	86.9	42	79.2	0.157
Can malaria be transmitted by blood transfusion? (Yes)	0	0.0	3	5.7	--
Is it necessary to go to the microscopist? (Yes)	192	89.7	49	92.5	0.548
Can malaria be cured with tablets? (Yes)	191	89.3	49	92.5	0.489
**Multiple choice questions**					
What are the Malaria symptoms?					
Fever	113	52.8	27	50.9	0.808
Headache	38	17.8	7	13.2	0.428
Chills	32	15.0	11	20.8	0.304
What happens if a person does not complete the treatment?					
Nothing	8	3.7	13	24.5	**0.000**
Can die	176	82.2	8	15.1	**0.000**
Who taught you what you know about malaria?					
Friends	142	66.4	18	34.0	**0.000**
Family	63	29.4	28	52.8	**0.001**
School	9	4.2	6	11.3	**0.044**
Responsible for malaria control					
Government	62	29.0	8	15.1	**0.040**
Health promoter	103	48.1	20	37.3	0.174
What can be done inside the house to prevent malaria?					
ITN	146	68.2	46	86.8	**0.007**
IRS	86	40.2	2	3.8	**0.000**
Cannot do anything	1	0.5	2	3.8	**0.041**
What can be done outside the house to prevent malaria?					
Avoid weeds, cut grass	74	34.6	3	5.7	**0.000**
Avoid standing water	85	39.7	2	3.8	**0.000**
Insecticide spraying	61	28.5	2	3.8	**0.000**
Cannot do anything	5	2.3	11	20.8	**0.000**
Not known - No answers	22	10.3	33	62.3	**0.000**

Responders in both MR and HR areas had variable knowledge on malaria preventive measures. Most participants knew about tablets that could cure malaria (MR = 89.3%; HR = 92.5%); however a small proportion (2.8%) of responders from MR areas believed that home treatments were more suitable to cure the disease, while in HR areas nobody believed this; and 1.9% indicated that malaria treatment was not necessary. With respect to completing drug treatment in MR areas, 82.2% knew that interrupting malaria treatment may lead to death, whereas in HR areas only 15.1% knew this (p =0.000). In both areas, the community health promoter was identified by responders as responsible for malaria control (MR = 48.1%; HR = 37.3%) (p = 0.174). For 66.4% of responders from MR areas, the main source of knowledge on malaria was friends, compared with 34.0% in HR areas. In contrast, in HR areas it was found that family played an important role (52.8%) as a source of knowledge on malaria, while in MR areas this was less prevalent (29.4%). This factor was significantly different between the responders of the two areas (p = 0.001). Surprisingly, few responders felt that the government or health promoters had taught them about malaria.

With regard to intervention measures for indoor prevention and vector control, a higher percentage of responders from HR areas (86.8%) mentioned the use of ITNs compared with responders from MR areas (68.2%). Despite MoH recommend to avoid indoor residual spraying (IRS) in areas with coverage of ITN, in MR areas, 40.2% of responders knew that indoor residual spraying (IRS) was a prevention measure compared with only 3.8% in HR areas (p < 0.000). Regarding outdoor vector control measures, a high percentage of people in MR areas knew that the presence of long grass (34.6%) and standing water (39.7%) should be avoided. Use of insecticide spraying (28.5%) in order to significantly reduce mosquito populations was recognized, whereas in HR areas knowledge of these three features was minimal (5.7, 3.8 and 3.8%, respectively). This knowledge was significantly different between the responders from the two areas. In HR areas 62.3% did not respond or did not know the availability of outdoor malaria prevention measures.

### Attitudes

In MR areas 45.8% of responders believed that getting malaria was normal, whereas only 11.3% in HR areas believed it was normal (Table 3) with significant differences (p = 0.000). In MR and HR areas, 89.3 and 77.4% of responders, respectively, had used anti-malarial drugs to treat malaria symptoms and as a useful means to kill the parasite (p = 0.021). Regarding diagnosis, 93.5% in MR areas and 71.7% in HR areas thought that the TBS was necessary to determine if a person had malaria (p = 0.000), additionally, 86.0% of responders from MR areas and 47.2% from HR areas considered that prescribed anti-malarials must be continued even after feeling better (p = 0.000); 29.4% in MR areas compared to 52.8% in HR areas admitted having purchased tablets without medical prescription (p = 0.001). Moreover, 9.8% of responders in MR areas compared with 22.6% in HR areas responded as if that malaria is a disease transmitted from a sick person to a healthy one through physical contact (p = 0.011).

**Table 3:** Attitudes about malaria

	**Medium risk**		**High risk**		
	**(n=214)**		**(n=53)**		
**Responded affirmatively**	**n**	**%**	**n**	**%**	**p-value**
Is getting malaria normal?	98	**45.8**	6	**11.3**	**0.000**
Does standing water facilitate transmission?	179	83.6	45	84.9	**0.823**
Can tablets cure malaria?	191	**89.3**	41	**77.4**	**0.022**
Is the blood smear necessary?	200	**93.5**	38	**71.7**	**0.000**
Is it needed to finish the treatment?	184	**86.0**	25	**47.2**	**0.000**
Have you had malaria more than once?	195	91.1	45	84.9	**0.179**
Have you bought tablets without a prescription?	63	**29.4**	28	**52.8**	**0.001**
Can physical contact transmit malaria?	21	9.8	12	22.6	**0.011**
Is it annoying to use bed nets?	39	18.2	14	26.4	**0.181**
Does the presence of mosquitoes bother you?	204	95.3	47	88.7	**0.068**

### Practices

**Figure 3:**
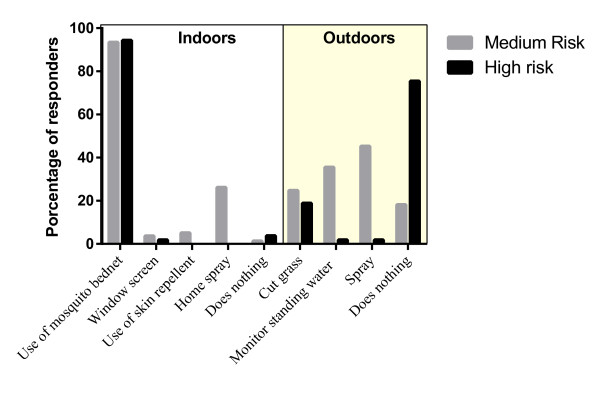
Practices to prevent malaria.

To control indoor malaria transmission, most responders in both regions (MR = 93.5%; HR = 94.3%) indicated the use of INT for malaria protection; further, 26.2% of responders from MR areas, but none from HR areas, reported using home spray (Figure 3). Regarding outdoor mosquito control, 35.5% of MR inhabitants regularly monitored the presence of standing water in their neighbourhoods compared to 1.9% in HR areas. Additionally, 45.3% of MR participants used insecticide home-spray compared with 1.9% in HR areas. In HR areas 75.5% of responders did nothing outside the home to prevent malaria. Regarding malaria diagnosis, at the onset of malaria symptoms 88.9% and 83% of responders in MR and HR areas, respectively, initially attended a local clinic with either a microscopist, physician or health promoter to perform the TBS. In MR areas, 29.0% of respondents consulted first with health services and 17.8% with the health promoter, whereas in HR areas these two alternatives were not consulted. About 75.7% and 98.1% of responders from MR and HR areas, respectively, reported that the place of malaria diagnosis was located less than one hour away from their households. In MR areas, 60.7% travelled to the point of diagnosis by foot, compared with 98.1% in HR areas; in MR areas 21.5% used a motor vehicle. The practices mentioned above were significantly different between the responders of the two areas (p < 0.050) (Table 4).

**Table 4:** Practices in malaria control

	**Medium risk**		**High risk**		
	**(n=214)**		**(n=53)**		
	**n**	**%**	**n**	**%**	**p-value**
**Yes/No question**					
Did you get a blood smear during your last malaria episode? (Yes)	170	79.4	47	88.7	0.123
**Multiple choice questions**					
Where to go when they feel sick?					
Physician, Nurse	62	29.0	0	0.0	**0.000**
Microscopists	90	42.1	44	83.0	**0.000**
Community health promotor	38	17.8	0	0.0	**0.000**
How long does it take to get to a malaria diagnosis post?					
Less than 1 hour	162	75.7	52	98.1	**0.000**
Between 1 to 2 hours	28	13.1	0	0.0	**0.005**
Means of transportation to the point of diagnosis					
Foot	130	60.7	52	98.1	**0.000**
Motor vehicle	46	21.5	0	0.0	**0.000**

### Association of malaria KAP and malaria incidence

Despite knowing that the use of an ITN prevents malaria transmission, no significant differences were found between this knowledge and having suffered malaria in the last year (p > 0.050). Likewise, no correlation was found between malaria prevalence in the three municipalities and the knowledge about use of IRS to reduce malaria transmission. This knowledge was related to malaria prevalence in MR areas (p < 0.050).

A high proportion of responders in both areas (93.5% in MR, and 94.3% in HR areas) reported using ITNs because they knew it prevented malaria transmission, however no significant differences were found between the use of ITNs and having malaria. Having ponds or standing water around the house was significantly associated with having suffered malaria over the last year (p < 0.050) in MR areas but not in HR areas: 36.4% of responders in MR areas and 4.8% in HR areas knew that avoiding standing water could reduce malaria transmission.

## Discussion

This study was conducted to identify the KAP related to malaria in endemic areas of Colombia. Significant differences in the level of education in the two group categories were found. HR areas showed a higher proportion of illiteracy than in MR areas (p = 0.000). Furthermore, the level of primary education was higher in HR areas (64.2%) than in MR (47.7%) (p = 0.031); however, the overall education level was higher in MR areas. Correlation between the level of formal education and malaria risk was not found, probably due to the overall high knowledge level in both areas. Most people in both regions stated that they had learned about malaria from their families (33.6%) or friends (59.0%). Previous reports indicating that the level of literacy has a direct correlation with best malaria practices [16,17], and that increasing the literacy level would serve as a protective factor against malaria morbidity [18,19]. Previous studies have also indicated that malaria knowledge was only marginally better in areas with higher education levels [20].

Similar to other reports [21-27], in this study, knowledge about malaria transmission mechanism was high. About 85% of the total responders knew that the bite of infected mosquito transmits the parasite to humans, and this could explain the high percentage of responders who knew about the benefits of ITN use; most of the study population (>90%) used an ITN. This extended use of ITNs appears to be a result of the aggressive distribution of ITNs by the joint NMCP and GFATM malaria control programmes.

Other studies carried out in Latin American and Caribbean countries have investigated knowledge of the role of mosquitoes in malaria transmission. In a previous study in Haiti, despite the high level of knowledge about the role of mosquitoes in malaria transmission (61.8%), poor ownership and use of ITNs was observed; no significant association was found between education and correct knowledge [13]. Likewise in Honduras, most participants in a KAP survey demonstrated general awareness of malaria and it transmission, but the study did not analyze the relationship between education level, malaria knowledge and disease prevalence [28]. In contrast in a study carried out in Mexico, malaria knowledge was poor; only 48% of the population associated malaria with mosquito bites, 99% of villagers had ITNs and 75.7% used them all year round [20]. In that study, the perceived benefit of IRS was associated with reduction in mosquitoes and other insects and pests, but only 3% associated it directly with prevention of malaria transmission. In Venezuela, 68.3% associated malaria with mosquito bites, however a high percentage (86.2%) of respondents refused to use ITNs [29]. However, these studies showed an overall perspective and do not establish regional differences.

About half of the population interviewed in the present study identified fever as a major malaria symptom, results that are consistent with other studies [21,22,30-34]. For most of the population (89%), fever with other classical malaria symptoms was an indication to visit a local clinic with a microscopist or other health officer. This was consistent with studies where communities with a good knowledge of the cause and manifestations of the disease seek early treatment [21,30], yet it is in contrast with others where >40% of responders delayed seeking malaria treatment [22,35]. In this context, it was surprising that about half of the responders in HR areas reported buying medicine without prescription, although this attitude was less prevalent (~30%) in MR areas. As reported in other studies, self-medication leads to late consultation, disease complications and possible emergence of drug resistance [36-42]. In the Mexican study, >40% of villagers were reported to self-medicate when a family member experienced a fever episode [20].

Another relevant finding was that people from Buenaventura and Tumaco, both MR areas, had greater knowledge and better practices against the disease, probably due to permanent malaria education programmes. Tumaco was the subject of previous interventions by a GFATM for malaria control in the borders of Peru, Ecuador, Colombia, and Venezuela. This appears to be in agreement with the finding that knowledge about IRS was significantly higher in the MR area than in HR area (40.2 *vs* 3.8%, p = 0.000), although overall results here are lower than those reported in a previous study carried out in Iran (64.6%) [17]. However research in Zimbabwe showed a significant relationship between malaria knowledge and the use of preventive measures in the house. The level of understanding of IRS was directly correlated to compliance with having the house fumigated [43].

The limitations of this study are associated with the design of data collection procedures. The cross-sectional design provides information about a particular point in time, but it is unable to determine rates of change or stochastic variation. In addition, the data were collected from households only; some information was not confirmed, for example, that related to ITN use.

## Conclusion

Despite high levels of knowledge in both regions, significant gaps relating to practices persist. There were significant differences in attitudes about buying medicine without a prescription, adherence to treatment and practices in both indoor and outdoor vector control prevention measures between respondents living in MR and HR areas. This study contrasts geographical regions of Colombia in their KAP on malaria and presents evidence that point to the need to develop educational programmes specific to each region based on the specific gaps found in this study. Education, control and elimination malaria programmes must take into account local characteristics in terms of the variables measured in this study for analysis, reporting and planning.

Finally, it is evident that communities in high and moderate transmission settings of Colombia are aware about transmission, symptoms, diagnosis and prevention of malaria. Nevertheless, Ministry of Health programmes must be able to correct misconceptions about malaria diagnosis and treatment as well as knowledge about mosquito control with focused health education initiatives.

## Competing interests

The authors declare that they have no competing interests.

## Authors’ contributions

SHV and MAR conceived and designed the study; PC, SH, MAR, AV, DF, and JG wrote the manuscript; YB and JG conducted data management and data analysis. All authors read and approved the final manuscript.

## Supplementary Material

Additional file 1 **The supplementary file is an analysis script in MATLAB to reproduce the results reported in the paper.** Data are available upon request, provided that the protocol to access, compliant with the funding agency, is followed. Click here for file
